# Joint-Preserving Surgery for Forefoot Deformities in Patients with Rheumatoid Arthritis: A Literature Review

**DOI:** 10.3390/ijerph18084093

**Published:** 2021-04-13

**Authors:** Koichiro Yano, Katsunori Ikari, Haruki Tobimatsu, Ayako Tominaga, Ken Okazaki

**Affiliations:** 1 Department of Orthopedic Surgery, Tokyo Women’s Medical University, Tokyo 162-8666, Japan; harukiryo0829@gmail.com (H.T.); kerokerokerori0812@gmail.com (A.T.); okazaki.ken@twmu.ac.jp (K.O.); 2 Institute of Rheumatology, Tokyo Women’s Medical University Hospital, Tokyo 162-8666, Japan; 3 Division of Multidisciplinary Management of Rheumatic Diseases, Tokyo Women’s Medical University, Tokyo 162-8666, Japan

**Keywords:** foot, joint-preserving surgery, orthopedic surgery, rheumatoid arthritis

## Abstract

The combination of first metatarsophalangeal joint arthrodesis and resection arthroplasty of all lesser metatarsal heads has been historically considered the golden standard treatment for rheumatoid forefoot deformities. However, as recent improved management of rheumatoid arthritis have reduced progression of joint destruction, the surgical treatments for rheumatoid forefoot deformities have gradually changed from joint-sacrificing surgery, such as arthrodesis and resection arthroplasty, to joint-preserving surgery. The aim of this literature review was to provide current evidence for joint-preserving surgery for rheumatoid forefoot deformities. We focused on the indications, specific outcomes, and postsurgical complications of joint-preserving surgery in this review.

## 1. Introduction

In the management of rheumatoid arthritis (RA), a paradigm shift has occurred in the past a few decades, produced by the introduction of effective and powerful antirheumatic medications and the progression of management strategies [1]. As a result, we have become able to control the disease activity of RA. However, the proportion of patients with ongoing symptoms in the foot joints is still high [2,3,4,5,6,7]. Once deformities occur in the feet, surgical intervention is the only option to completely correct them. The combination of first metatarsophalangeal (MTP) joint arthrodesis and resection arthroplasty of all lesser metatarsal heads has been historically recognized the golden standard treatment for rheumatoid forefoot deformities [8]. However, as recent improved treatment of RA have reduced progression of joint destruction, the surgical treatments for rheumatoid forefoot deformities have gradually changed from joint-sacrificing surgery, such as arthrodesis and resection arthroplasty, to joint-preserving surgery, especially in Japan [9,10,11,12,13,14,15,16,17]. As many orthopedic surgeons in Japan not only perform surgeries but also provide medical treatments for patients with RA, they are well aware of the dramatic progression in medications and treatment strategies for RA. As a result, they hesitate to sacrifice less damaged joints.

Many surgical techniques are available that are aimed at preserving joints, including various types of osteotomy and soft tissue reconstruction. The aim of this literature review was to provide current evidence and detailed procedures for joint-preserving surgery for rheumatoid forefoot deformities. The review includes scientific articles written in English, available in the PubMed/Medline database, and published before 25 February 2021. We hope that this literature review serves as a resource for surgeons for choosing the optimum surgical methods to achieve the best practice and restore foot health.

## 2. Joint-Preserving Surgery

The concept of “joint-preservation” was introduced by Hanyu et al. in 1997. They started joint-preserving surgery to preserve the function of the MTP joints as they recognized its function as important for gait even in patients with RA [18]. Thus, joint-preserving surgery is defined as forefoot surgery that preserves the MTP joints. It consists of a combination of metatarsal osteotomy and soft tissue reconstruction. Bhavikatti et al. suggested that the purpose of joint-preserving surgery was not to create a normal foot, but to correct deformity, alleviate pain, and improve function [10].

### 2.1. Indications

Joint-preserving surgery is performed in patients with RA who suffer from pain and deformity of the forefoot or plantar callosities despite conservative treatment.

The indication criteria for destruction of the MTP joints vary according to previous reports and are as follow: Larsen grades 2 [12,19], 3 [13,14,18], and 4 [16,20]. The Larsen scoring system is a radiological method for grading the severity of joint destruction in patients with RA [21]. On the other hand, Hirao et al. performed joint-preserving surgery for any joint destruction, except for pencil deformities of the metatarsal head [11,22].

As for radiographic assessment, a hallux valgus angle (HVA) > 15° and intermetatarsal angle between the first and second metatarsals (IMA) > 15° [23], as well as HVA > 25° [11] and IMA > 10° [16,20] were indicated as inclusion criteria for joint-preserving surgery.

### 2.2. First Ray

#### 2.2.1. Modified Scarf Osteotomy

**Figure 1:**
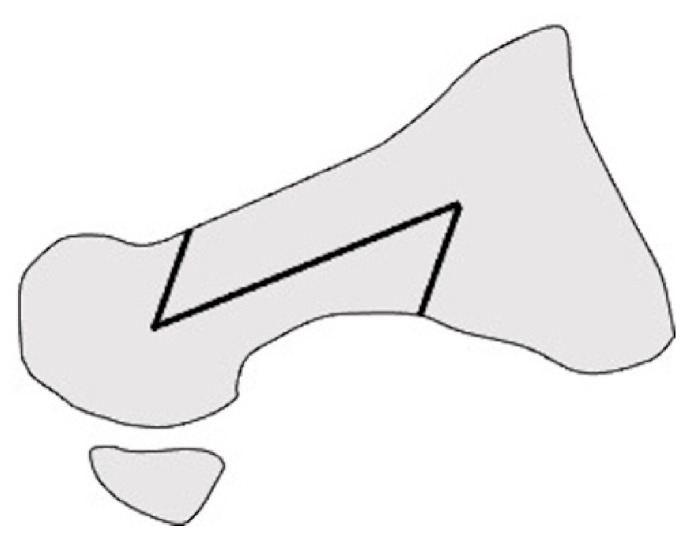
Scarf osteotomy in lateral view.

Scarf osteotomy is a z-shaped osteotomy of the first metatarsal (Figure 1) [24]. Scarf is a carpentry term that describes the beveling of the ends of two pieces of wood such that securely fastening them creates overlap, leading to one continuous piece [25].

Barouk et al. performed the modified scarf osteotomy suggested by Maestro in which a notch was created via medial extension of the cephalic part of the osteotomy, the plantar fragment was displaced laterally, and the distal end of the proximal fragment was then fitted into the notch [26]. They reported outcomes in 55 cases of joint-preserving surgery using this modified scarf osteotomy and the Weil osteotomy for the lateral metatarsals [9]. Over an average of 6 years of follow-up, maintenance of satisfactory correction was found in 95% of the cases. There was only one case of failure which required revision surgery for arthrodesis of the first metatarsal. They emphasized that the most important component of correction was the shortening of the first metatarsal. In addition, they warned that excessive lateral translation of the distal bone fragment led to overcorrection, especially in cases with a narrow IMA [9].

Bhavikatti et al. reviewed 66 feet that underwent scarf osteotomy of the first metatarsal with a Weil osteotomy of the lesser metatarsal. Over 87% of patients subjectively reported their outcome as excellent or good, and the mean American Orthopedic Foot and Ankle Society Score (AOFAS) score increased from 39.8 to 88.7 after surgery [10].

**Figure 2:**
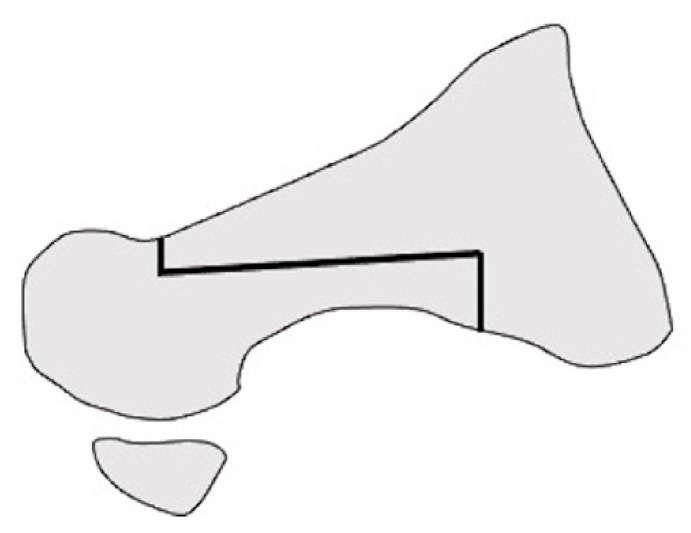
Modified scarf osteotomy in lateral view.

Hirao et al. performed another modified scarf osteotomy in which the osteotomy was done parallel to the plantar aspect, and both ends of the bone fragments were partially resected (Figure 2). Moreover, an interposition technique for the medial capsule of the first MTP joint was added [11,22,27]. The medium-term outcomes in 108 cases of osteotomy indicated significant improvements on both the Japanese Society for Surgery of Foot (JSSF) scales and radiographic assessments at the last follow-up. Hallux valgus (HV) recurrence occurred in 16% of patients with relatively high preoperative IMA (average, 17.7°). The study also found that the preoperative disease activity score of RA significantly correlated with the JSSF scale and HVA at the final follow-up. This suggests that higher disease activity is associated with both clinical and radiographic outcomes [11].

#### 2.2.2. Lapidus Arthrodesis

Lapidus arthrodesis is the first tarsometatarsal (TMT) joint fusion procedure, introduced as a surgery for idiopathic HV in 1934 [28]. In this procedure, after osteotomy of the TMT joint, the first metatarsal is abducted to correct the HV while preserving the first MTP joint.

Shi et al. evaluated a modified Lapidus technique in 21 rheumatoid HV deformities. They described satisfactory pain relief, footwear comfort, satisfaction with the outcome of the surgery, and radiographic improvement after surgery. In addition, there was a strong correlation between splay deformity and the recurrence of HV [29].

Popelka et al. performed Lapidus arthrodesis in 143 patients with RA to treat hypermobility of the TMT joint leading to the development of HV. To assess hypermobility, they examined the preoperative anteroposterior radiographs of the weight-bearing and non-weight-bearing feet with measurement of the IMA. Moreover, weight-bearing lateral X-rays were focused on the asymmetry of the TMT joint gap [30]. Hypermobility of the TMT joint was found in 64.3% of cases. In 10 cases, after surgery, the IMA did not reduce to normal levels due to insufficient resection of the TMT joint; therefore, they suggested that appropriate bone resection is essential for positive postoperative outcomes with Lapidus arthrodesis [23].

**Figure 3:**
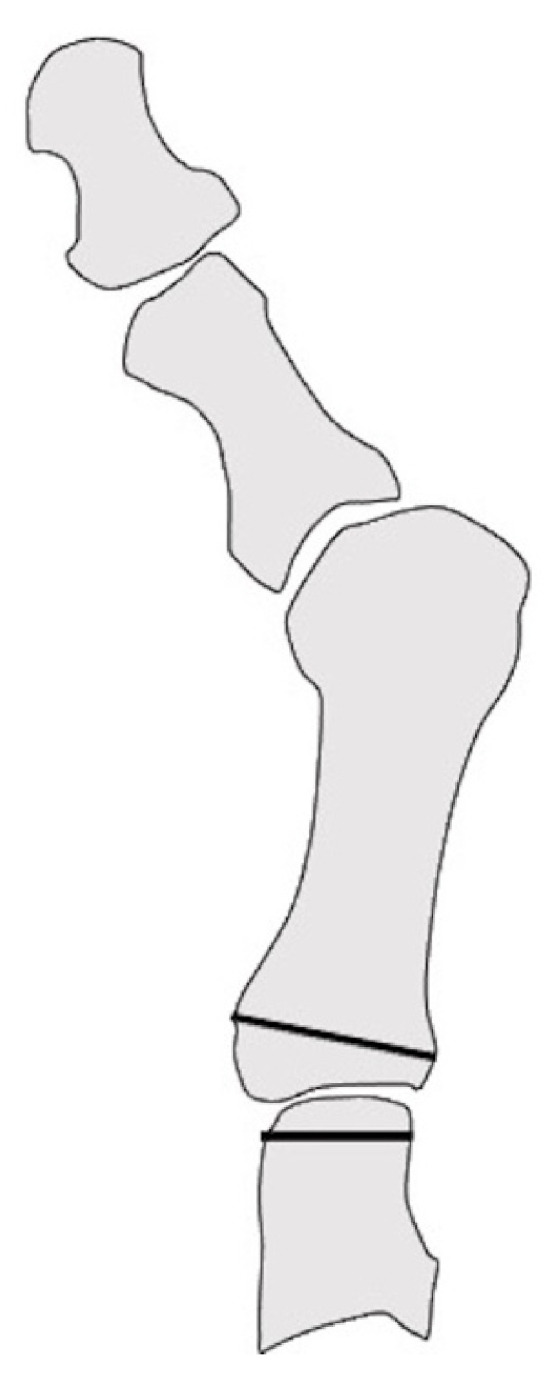
Modified Lapidus arthrodesis in anteroposterial view.

Long-term outcomes of the modified Lapidus arthrodesis, in which the first metatarsal was abducted, shortened, and supinated after osteotomy (Figure 3), combined with proximal shortening osteotomy of the lesser metatarsals, were reported by Niki et al. [13]. The surgery was performed on 57 RA patients in clinical remission, with an average of 76.6 months of follow-up. The JSSF score and radiographic assessments significantly improved at the last follow-up. No patient experienced obvious recurrence of the deformity or symptomatic callus formation. They suggested that one of the advantages of Lapidus arthrodesis is that correction loss due to postoperative changes in TMT joint alignment can be avoided [13,14].

#### 2.2.3. Proximal Rotational Closing-Wedge Osteotomy

**Figure 4:**
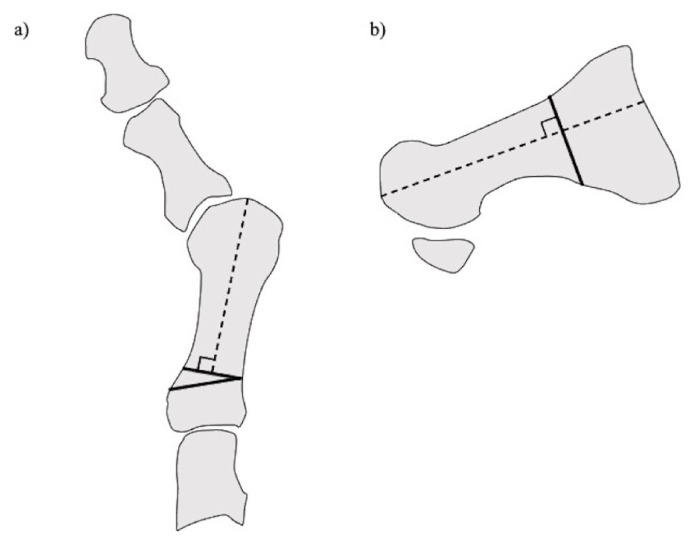
Proximal rotational closing-wedge osteotomy. (**a**) Anteroposterial view. (**b**) Lateral view.

Authors in this article developed proximal rotational closing-wedge osteotomy of the first metatarsal, in which the distal aspect of the wedge was vertical to the axis of the first metatarsal (Figure 4). The benefits of the osteotomy are as follows: (1) large correction due to proximal osteotomy; (2) correction of the pronation deformity of the first metatarsal by supinating the distal fragment of the first metatarsal; (3) easy shortening of the first metatarsal according to the amount of shortening of the lesser metatarsals; and (4) easy correction as planned preoperatively by placing osteotomy surfaces in contact without fluoroscopy [16,20].

**Figure 5:**
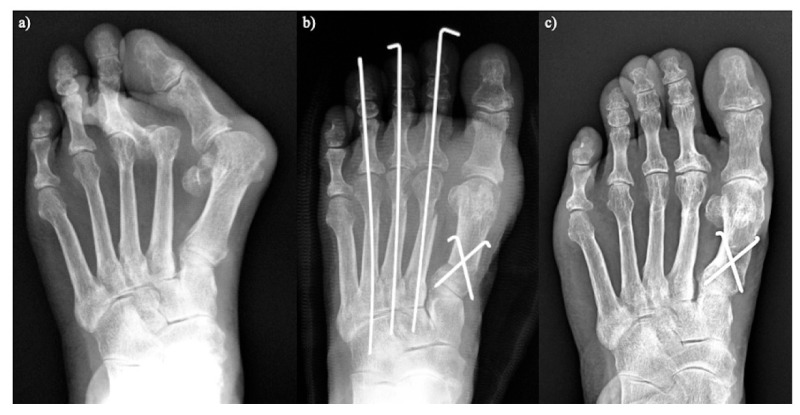
Proximal rotational closing-wedge osteotomy. (**a**) Preoperative. (**b**) Two weeks after surgery. (**c**) Five years after surgery.

We assessed the long-term outcomes, including patient-reported outcome measures and radiographic evaluations, of proximal rotational closing-wedge osteotomy in 105 rheumatoid feet with a minimum follow-up of 5 years. Patient-reported outcome measures were evaluated using the self-administered foot evaluation questionnaire (SAFE-Q). All SAFE-Q subscales and radiographic values improved significantly over an average follow-up of 6 years. Recurrence of HV occurred in 10.5% of cases. The estimated survivorship of joint-preserving surgery with proximal rotational closing-wedge osteotomy of the first metatarsal and shortening oblique osteotomies of the lesser toes, at 7 years, with reoperation as the endpoint, was 89.5% (Figure 5) [20].

#### 2.2.4. Other Osteotomies

Thordarson et al. performed chevron osteotomy in 13 cases with well-preserved joint space and no active preoperative signs of inflammation, but approximately 85% of cases developed a HV deformity again or inflammatory erosions, with an average time to failure of 2 years [31].

Nagashima et al. reported the outcomes of a modified Hohmann osteotomy [32] combined with a Helal osteotomy [33] in the lesser toes of 47 rheumatoid feet. They reported that HVA and IMA improved significantly at final follow-up, and all patients were satisfied with the modified Hohmann procedure [12].

Takakubo et al. used a modified Mann method in 11 cases that combined proximal osteotomy of the first metatarsal and soft tissue reconstruction. The procedure showed satisfactory clinical and radiographic outcomes. Joint degeneration scores of the first MTP joint as per the modified Sharp methods [34] and Larsen grades [21] were also examined. Only the postoperative joint space narrowing score showed a slight decline. Although the recurrence of HV involving a HVA of more than 25° was relatively high (27%), this may be due to the small number of cases [15].

Chao et al. performed several osteotomies (Ludloff, scarf, and chevron) to preserve the first MTP joint in 37 rheumatoid feet. The AOFAS and visual analogue scale (VAS) pain scores improved significantly in this series. In contrast to Takakubo et al. [15], the joint degeneration scores of the first MTP increased postoperatively.

Jo et al. compared a fixation technique involving the modified Ludloff osteotomy using two screws with a plate augmentation along with two screws in 31 rheumatoid feet. Correction loss at the osteotomy site occurred only in patients who underwent fixation with two screws, without the plate [19].

### 2.3. Lateral Rays

Most osteotomies of the lesser metatarsals aim to shorten the metatarsal and elevate the metatarsal head dorsally. This shortening enables correction of the dorsally dislocated lesser toes, and elevation reduces the risk of callosity recurrence.

#### 2.3.1. Shortening Oblique Osteotomy of the Metatarsal Neck

**Figure 6:**
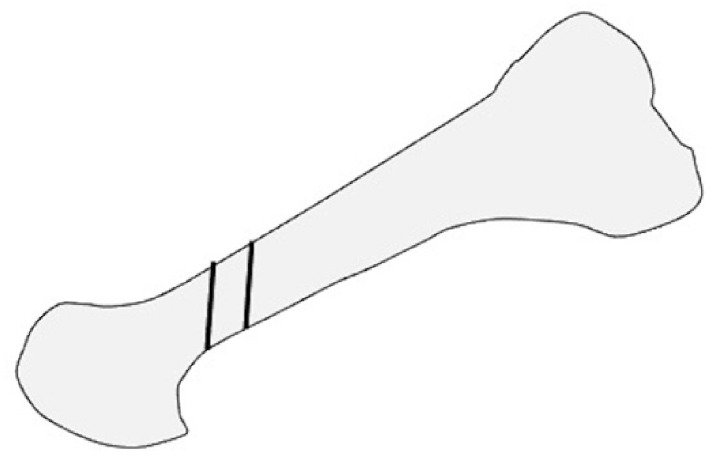
Shortening oblique osteotomy of the lesser metatarsal in lateral view.

Shortening oblique osteotomy (SOO) of the metatarsal neck was first performed by Hanyu et al. in 1997 (Figure 6). Although a simple oblique osteotomy of the metatarsal neck had been developed by Helal et al. [33], Hanyu et al. modified it by cutting the metatarsal neck twice in a parallel manner to shorten metatarsal length and to elevate the metatarsal head. After correcting the dislocation of the MTP joints of the lesser toes, the joints and osteotomy sites were temporarily fixed using a single Kirschner wire (K-wire) [18]. We have reported satisfactory short- and long-term outcomes after SOO with a modification involving a ligature around bone fragments to hold them together to avoid nonunion or malunion along with proximal rotational closing-wedge osteotomy of the first metatarsal [16,20,35]. Horita et al. fixed the osteotomy site with a screw instead of a K-wire and described good clinical outcomes with SOO [36].

#### 2.3.2. Weil Osteotomy

**Figure 7:**
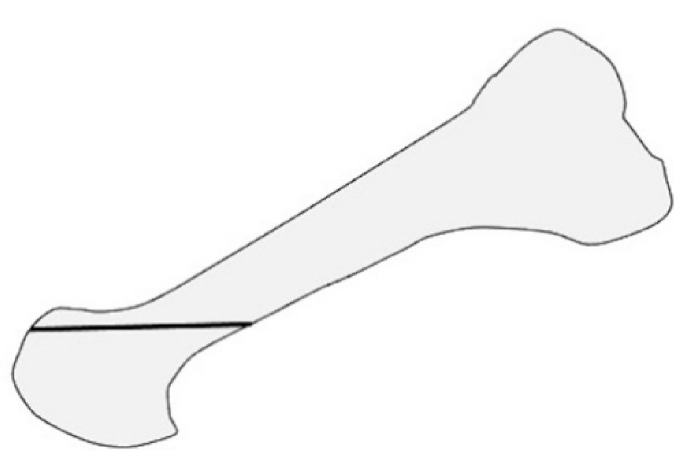
Weil osteotomy of the lesser metatarsal in lateral view.

Weil osteotomy is cut from the dorsal surface of the metatarsal head to the proximal plantar surface along the metatarsal shaft, while attempting to be parallel to the ground, which allows a fixed amount of shortening of the metatarsal (Figure 7). After shortening was completed, a screw was used to fix the two fragments [37,38].

Barouk et al. modified Weil osteotomy by additional removal of a thin trapezoidal bone to elevate the plantar aspect of the metatarsal head. For preoperative assessment, they set the metatarsal shortening point (MS point), which is the location of the most proximal part of the base of the proximal phalanx of the most impaired ray. The amount of metatarsal shortening was determined as the distance between the MS point and the distal end of the metatarsal head. In their study, average shortening amounted to 13 mm. This study described failed joint preservation of the lesser MTP joint, resulting in secondary surgery occurring in only 2% of all cases. They suggested that the shortening should reach the MS point to achieve successful correction of the deformities [9].

Bhavikatti et al. examined 66 feet that underwent joint-preserving surgery combined with a scarf osteotomy of the first ray and Weil osteotomy of the lesser metatarsal. As described above, the patients showed good clinical and radiographic results [10].

#### 2.3.3. Offset Osteotomy

Offset osteotomy involves shortening of the metatarsal bone and shifting the metatarsal head medially for lesser MTP joint deformities, as reported by Owaki et al. in 2003. After the osteotomy, two notches were made in the cortical bone at the distal end of the proximal stump. The proximal end of the metatarsal head was inserted into the notch to achieve shortening and shifting of the lesser metatarsals [39].

**Figure 8:**
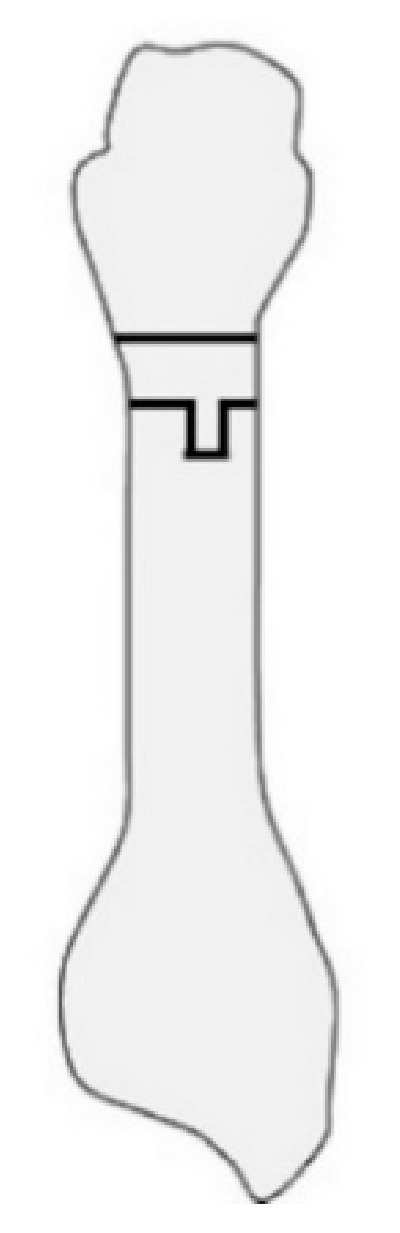
Offset osteotomy of the lesser metatarsal in anteroosterial view.

Hirao et al. changed the direction of shift of each metatarsal head to reconstruct the transverse arch (Figure 8). This modified offset osteotomy showed satisfactory clinical and radiographic outcomes in 80 patients with rheumatoid forefoot deformities. Recurrence of dorsal dislocation and subluxation of the lesser MTP joints occurred in some cases. In the recurrence group, 67% showed varus hindfoot preoperatively, and most of the preoperative valgus hindfoot cases involved midfoot ankyloses. They suggested that surgeons had to consider the possibility of recurrence of lesser MTP joint deformities in cases of varus hindfoot and midfoot ankyloses [17].

#### 2.3.4. Proximal Oblique Shortening Osteotomy

**Figure 9:**
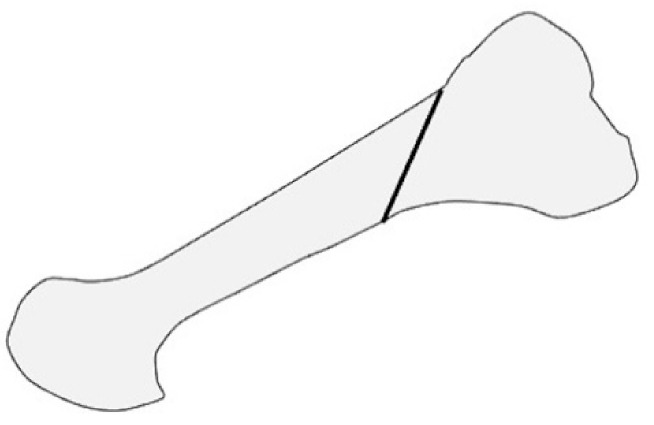
Proximal oblique shortening osteotomy of the lesser metatarsal in lateral view.

Niki et al. developed shortening oblique osteotomies of the bases of the second, third, and fourth metatarsals combined with a modified Lapidus arthrodesis of the first TMT joint and a modified Coughlin osteotomy of the fifth metatarsal. Oblique osteotomy was performed at an angle of 30° to the longitudinal axis of the metatarsal (Figure 9). After the distal fragment was displaced dorsally and proximally to shorten and elevate the metatarsal, the osteotomy site was fixed using a screw. No obvious recurrence of deformity or symptomatic callosity occurred after surgery in 57 cases [13].

### 2.4. Post-Operative Protocol

The time at which weight-bearing is started varies according to past reports: heel gait immediately after surgery [9,16], heel gait 1 week after surgery [19], full weight-bearing two or three weeks after surgery [12,15,18,22], partial weight-bearing 3 weeks after surgery [13,14], and full weight-bearing 6 weeks after surgery [10,23]. Most authors allowed patients to wear an orthosis or a specific shoe during walking.

K-wires inserted from the toes to fix the osteotomy site of the lesser metatarsals were usually removed 3 weeks after surgery [13,14,15,16,18]. Hirao et al. found that early introduction of range of motion (ROM) exercises following the removal of K-wires 2 weeks after surgery increased the ROM of the lesser toes 6 months after surgery, when compared to the non-exercise group [40].

### 2.5. Changes in Plantar Pressure after Joint-Preserving Surgery

Foot deformities cause abnormal plantar pressure distribution. In RA patients, as the magnitude of the deformity is often severe, impaired distribution leads to walking disability. In particular, high plantar pressure is associated with callosity and pain during walking [41]. Therefore, correction of the deformity by surgery is very important to obtain a close-to-normal plantar pressure distribution. The key to a successful outcome after joint-preserving surgery is to achieve stable realignment of the first ray in order to increase weight-bearing along the medial column of the foot and minimize stress on the lateral metatarsal heads [10].

Ebina et al. evaluated the effects of joint-preserving surgery (modified scarf osteotomy of the hallux and off-set shortening osteotomy of the lesser toes [22]) on plantar pressure distribution by comparing distribution in healthy controls, deformed non-operated feet, and feet that underwent resection arthroplasty. Their joint-preserving surgery increased plantar pressure distribution of the first MTP joint and decreased that of the second to fifth MTP joints. Moreover, joint-preserving surgery tended to show medial loading and high first MTP joint pressure, similar to that seen in healthy controls [42].

Lee et al. assessed changes in plantar pressure distribution after SOO of the lesser metatarsals combined with Swanson implant arthroplasty of the first MTP joint. The peak pressure at the 1st interphalangeal joint and the 2nd and 3rd MTPs decreased. On the other hand, the peak pressure at the midfoot increased, indicating a decrease in the postoperative longitudinal arch. They suggested that this phenomenon was caused by loss of the truss mechanism after shortening of the metatarsal [43]. Lee et al. also attempted to clarify the surgical indications for rheumatoid forefoot deformity by comparing the difference in plantar pressure distribution between feet with and without scheduled surgery. They found that the combined assessment of HVA, and Δ pressure, representing the difference between maximum peak pressure and minimum peak pressure, was useful as a surgical indicator [44].

Shimoda et al. examined the effects of forefoot arthroplasty, including joint-preserving surgery, on plantar pressure. The average plantar pressure under the second to fifth metatarsal heads declined after surgery, as in other reports [45].

The authors of this article revealed that the movement distance of the center of pressure was extended after joint-preserving surgery. We propose that this result means that the toes were able to touch the ground after surgery, and thus improved the push-off mechanism by the toes [46].

### 2.6. Complications

#### 2.6.1. Recurrence of Deformity

The recurrence rate of HV deformities after joint-preserving surgery was 0–27% Ref. [10,11,13,15,18,20]. However, the definition of recurrence was slightly different for each author. Recurrence is the most common complication associated with HV surgeries [47]. Preoperative severe deformities, such as IMA > 17.7° [11] or HVA > 45° [10], are a reason for recurrence. Some researchers have reported that hindfoot malalignment is also a reason for recurrence after HV surgery in RA patients [48,49,50,51]. However, in some other reports, there was no significant relationship between hindfoot or midfoot alignment and the recurrence of HV deformity [11,20]. Thus, further research is needed in the future to clarify the associations between malalignment of the midfoot and hindfoot, and the risk of recurrence of HV deformity. In the lesser toes, the recurrence rates of deformity were reported to be 0–30% [10,11,13,18,20].

#### 2.6.2. Delayed Wound Healing

Delayed wound healing often occurs after foot surgery because of the thin soft tissues and poor circulation in the feet. In particular, reconstructive surgeries for rheumatoid forefoot deformity require multiple incisions and simultaneous operative procedures to correct deformities, which may also increase the risk of problems in wound healing. The rate of delayed wound healing after joint-preserving surgery is described as 2.2–20% [11,12,13,18,20,23]. The authors in the present study used multiple regression analysis to reveal that longer operative time was a risk factor associated with delayed wound healing in patients with RA undergoing forefoot surgery; however, the cases in this study included not only joint-preserving surgeries but also joint-sacrificing surgeries [52].

#### 2.6.3. Surgical Site Infection

Although the occurrence rate of delayed wound healing is relatively high, that of surgical site infections (SSI) is low (0–1.8% [10,11,13,23]). However, the patients with SSI usually require the removal of osteosynthetic materials in addition to antibiotic therapy.

#### 2.6.4. Hallux Varus

Hallux varus is a major complication after HV surgery, leading to patient dissatisfaction. The occurrence rate of hallux varus deformity after joint-preserving surgery for rheumatoid forefoot deformities ranges from 3.8–9.0% [11,20]. Overcorrection to avoid recurrence of HV deformity is a major cause of hallux varus deformity.

#### 2.6.5. Recurrence of Plantar Callosity

Many patients with RA who decide to undergo forefoot surgeries hope for release from the severe pain of plantar callosities. Nevertheless, the recurrence rate of callosities is reported to range from 0 to 17% after joint-preserving surgery [12,13,18,20]. The key to reducing the rate is the construction and maintenance of a smooth arc [53] of the metatarsal heads after surgery [9,13,23].

#### 2.6.6. Nonunion at Osteotomy Sites

Most joint-preserving surgeries require metatarsal osteotomies. However, non-union rates at the osteotomy site are as low as 0–5.0% [10,11,13,18,20,23]. Moreover, most patients do not complain of pain, even if postoperative radiographs show non-union at the osteotomy sites. We found that a combination of dripping normal saline on the oscillating saw, ligating the osteotomy sites, and suturing the periosteum at the osteotomy sites decreased the frequency of delayed union after SOO [35]. Moreover, long-term outcomes after SOO revealed that all osteotomy sites achieved bone union over 5 years of follow-up [20].

#### 2.6.7. Restricted Range of Motion

Around half the patients had toe stiffness after joint-preserving surgery [14,54]. Hirao et al. investigated the effects of passive range of motion (ROM) exercise initiated 2 weeks after joint-preserving surgery, and reported that the exercise increased passive ROM at 6 months after surgery, when compared to the non-exercise group [40].

## 3. Joint-Preserving Surgery vs. Joint-Sacrificing Surgery

It is unclear which procedure, joint-preserving or joint-sacrificing, is superior for rheumatoid forefoot deformities. Schrier et al. reported that there was no clinical difference between joint-preserving surgery and joint-sacrificing surgery in their randomized trial, but their sample size was very small [55]. Large-scale randomized trials are needed to clarify this issue.

Some authors compared joint-preserving surgery and joint-sacrificing surgery in nonrandomized studies. Fukushi et al. reported a comparative study of joint-preserving surgery and joint-sacrificing surgery performed by different surgeons. The study described that after joint-preserving surgery, the function of the hallux and alignment of the lesser toes on objective assessments were superior to those after joint-sacrificing surgery [56]. Ebina et al. compared outcomes between joint-preserving surgery and joint-sacrificing surgery, focusing on SAFE-Q scores. Some subscales of the SAFE-Q were significantly improved in the joint-preserving surgery group than in the joint-sacrificing surgery group [57]. The same authors also compared the postoperative plantar pressure distribution of these two procedures in another study. Joint-preserving surgery postoperatively resulted in a significantly higher first MTP joint, lower second-third MTP joints, and lower fourth–fifth MTP joint plantar pressure, when compared to joint-sacrificing surgery [42]. Shimomura et al. compared resection arthroplasty and joint-preserving surgery based on shared decision-making methods. Better objective assessment was seen 12 months after surgery with joint-preserving surgery than resection arthroplasty. They also found differences in the postoperative time course of the SAFE-Q. The scores in joint-preserving surgery gradually improved during the study period; however, the scores in resection arthroplasty decreased 3 months after surgery and then improved to reach a plateau 6 months post-operatively [58].

## 4. Limitations

There are some limitations to this review article. First, statistical analyses were not performed to compare the outcomes of each procedure because this article is not a systematic review. Second, as only the Pubmed/Medline database was used to search relevant research, we may not have captured all the articles related to joint-preserving surgery.

## 5. Conclusions

There are many kinds of surgical procedures for joint-preserving surgery, as described in this review article. Although the short- and mid-term clinical and radiographic outcomes of joint-preserving surgery are satisfactory in general, few reports have described long-term outcomes, and further research is needed.

## Data Availability

All data is available through cited publications.
